# Tattlers

**Published:** 1890-11-15

**Authors:** 


### Passing Topics.

### TATTLERS.

The "Training of a Nurse" in this month's *Scribner* is yet another contribution to a theme which bids fair to be worn threadbare before it is laid aside. We confess to being of opinion that the subject is one which is far from well suited to the pages of a magazine for general readers, and we have often asked ourselves, what is the real object certain women put before themselves when they sit down to write about nursing, and to serve up for all comers an olla podrida of daily life in hospitals, seasoned with many details which were much better left un-narrated.

The writer of the paper under present notice tells us that "what is required is that a probationer shall be receptive." We do not dissent from this. Every learner is required to exhibit this same quality and it is considered especially a feminine characteristic; but there is another disposition the sex is credited with—the very antithesis of the first—and which we venture to believe a nurse of all people would do well to restrain. It is not necessary, or even seemly, that a nurse should take the world into her confidence concerning her achievements in the ward, and we have a strong conviction that this constant blabbing, this laying-bare of essentially private proceedings, this drawing back of the veil and exposing sad scenes of pain and death to every eye, is little calculated to raise nurses in the esteem of their readers, whatever the vanity of the writers may induce them to believe; and must be singularly offensive to the large body of their fellow-workers who, to their infinite credit, go about their work in silence, actuated only by the promptings of duty.

If anything were needed to enforce a criticism for which we claim no merit of originality—it is on many people's tongues—we have the difficulty such writers evidently experience in maintaining a grave countenance though they are dealing with a very serious subject. As they proceed, it almost seems as if their aim were to show how lightly they can treat the most painful episodes. We may be old-fashioned, but it appears to us in the worst taste to write jocosely that "the patients have a way of dying at night in spite of the best efforts of the nurse to keep them alive until morning." Has the woman who penned these lines persuaded herself she is less mortal than the patients she tends, that she can write thus thoughtlessly of the solemn moment which must come to us all? Perhaps even more objectionable are the particulars given of "stretcher" cases, and the tone of the observations upon operations. "In running an operation," we are told, "a nurse always aims at having it go off without a hitch, and sometimes it does, sometimes not." Of course, this is American literature, but it is offered to English readers; and we fear that, apart from differences of expression, many of the effusions so common at home are just as flippant, and one might almost say, just as gross. But were the narrative ever so well written, where we may fairly ask, is the use of it? Educational value it has none; apart from the opening page it is a mere piece of gossip. It would be a feeble production to put before any class of readers; in a family magazine it is clearly out of place.

We should not do the large majority of nurses the flagrant injustice of thinking that, individually, they need either reproof or warning in this connection. Nevertheless, they, as a body, are involved in the reflections which arise. Nurses, if they desire their calling to maintain its high place in public esteem, need not only to practice individually certain virtues, of which reticence is one, but they must create an *esprit* capable of exercising collective control, and how potent that can be made will become evident if they study the unwritten laws which govern the medical profession. No class of workers better deserves the recognition nurses have lately attained, but if they would hold the position the world is ready to accord them they must steadfastly set themselves to discountenance by every means in their power those literary efforts, which show the writers to be insensible to the best principles of their order, and indifferent to the requirements of decorum.

Agricola.

